# Not just a by‐product: circular DNA molecules derived from V(D)J recombination are linked to worse prognosis in B‐cell leukemia

**DOI:** 10.1002/1878-0261.70134

**Published:** 2025-09-25

**Authors:** Davide Pradella, Andrea Ventura

**Affiliations:** ^1^ Cancer Biology and Genetics Program Memorial Sloan Kettering Cancer Center New York New York USA

**Keywords:** BCP‐ALL, ecDNA, excised signal circles, genome instability, V(D)J recombination

## Abstract

Excised signal circles (ESC) are circular DNA molecules generated during T‐ and B‐cell maturation. Previously considered biologically inert, recent work by Gao *et al*. now show that ESCs can replicate and accumulate in healthy lymphocytes. Moreover, the authors link higher levels of ESCs to an increased risk of relapse in B‐cell leukemia patients and propose that this phenomenon is due to the unique ability of ESCs to induce genome instability.

## Graphical abstract

Gao *et al*. report that circular DNA molecules created as by‐products of V(D)J recombination during lymphocyte maturation (ESCs) can replicate and be retained for much longer than previously thought in healthy cells. In BCP‐ALL cells, increased ESC abundance correlates with a greater chance of relapse likely mediated by their ability to induce genome instability.

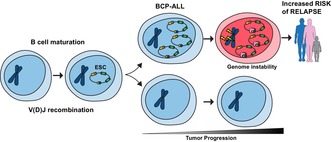

**Abbreviations**
BCP‐ALL: B‐cell precursor acute lymphoblastic leukemia
cRSS: cryptic recombination signal sequences
DSB: double‐strand breaks
ecDNA: extrachromosomal DNA
ESC: excised signal circles
NHEJ: nonhomologous end joining
RSS: recombination signal sequences

In both healthy and cancer cells, a fraction of the human genome can be found as extrachromosomal circular DNA molecules that are by‐products of both regulated and random processes. Are they bystanders or do they play an active role in disease?

Oncogene‐containing extrachromosomal DNAs (ecDNAs) are large (0.5–5 Mbp) circular DNA molecules thought to originate via accidental DNA double‐strand breaks (DSBs) followed by ligation‐mediated circularization. These ecDNAs have been conclusively shown to contribute to malignant transformation, tumor progression, and therapeutic resistance in cancer patients [1, 2, 3]. They can accumulate in large numbers because of the strong fitness benefit that the gene(s) they carry confer to cancer cells, combined with their ability to segregate asymmetrically due to the lack of centromeric sequences [4]. However, oncogene‐carrying large ecDNAs are not the only circular DNA molecules found in mammalian cells. In fact, the formation of circular DNA molecules is a physiological consequence of apoptosis [5] and B‐ and T‐cell receptor diversification [6, 7].

Circular DNA molecules known as excised signal circles (ESC) are generated as by‐products of V(D)J recombination, a critical step required for B‐ and T‐cell antigen diversity. The process is mediated by the recombination‐activating gene proteins (RAG1 and RAG2), which recognize recombination signal sequences (RSS) sites oriented in opposite directions in antigen receptor loci and promote recombination among RSS sites to form a functional receptor gene [8] (Fig. 1A).

**Fig. 1:**
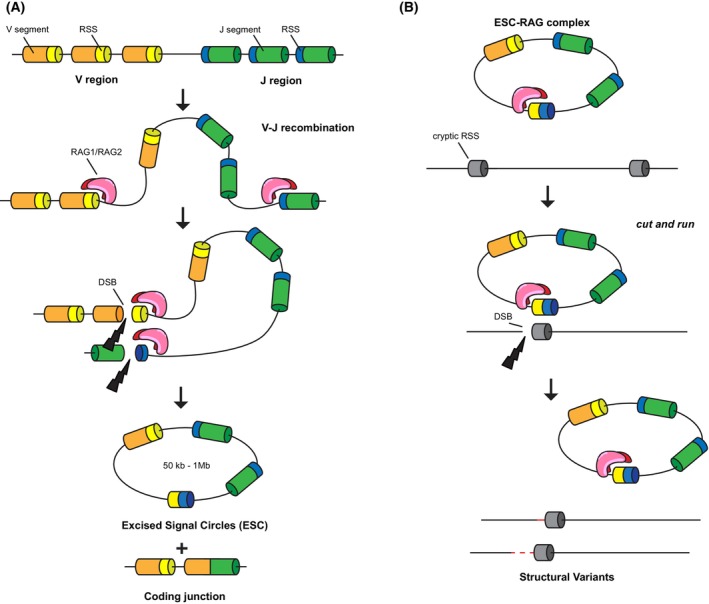
Excised signal circle (ESCs) formation and genome instability. (A) ESC DNA molecules are generated as by‐products of V(D)J recombination (VJ recombination is shown). The entire process is mediated by the recombination‐activating gene (RAG) proteins (RAG1 and RAG2; in pink/red), which randomly recognize recombination signal sequences (RSS) sites oriented in opposite directions (in yellow and blue) in the V (in orange) and J region (in green) of the locus. In addition to recognizing RSS sites, the RAG complex promotes the formation of double‐strand breaks (DSBs), which are repaired by the nonhomologous end joining (NHEJ) pathway, thus creating a novel coding junction between a V and a J segment. (B) The signal junction formed in the ESC is also recognized by the RAG proteins, which can mediate the recognition of cryptic RSS sites in the genome. Once recognized, the ESG‐RAG complex is able to induce the formation of DSBs that, if not properly repaired, lead to the formation of structural variants (insertion, deletion, rearrangements).

ESCs range in size from a few hundred nucleotides to 1 Mbp and—like oncogenic ecDNA—lack centromeric regions. Due to the absence of known functional elements, ESCs were, until now, thought to be short‐lived by‐products of lymphocyte maturation that are rapidly lost as cells divide. In a surprising turn of events, Gao *et al*. now show that ESCs can actively replicate and persist across multiple rounds of cell division in mature lymphocytes [9]. Furthermore, they link the increased abundance of ESCs in B‐cell precursor acute lymphoblastic leukemia (BCP‐ALL) to a greater risk of disease relapse.

But how can these circular DNA molecules affect tumorigenesis, given that they do not encode for known genes? The key may lie in their ability to associate with the RAG proteins, the same proteins that are responsible for their formation in the first place. Previous work indicated that ESCs might promote genome instability through reintegration at cryptic sites similar to the RSS sites (cRSSs) [10]. This reintegration process is mediated by the RAG proteins, which need to be loaded onto the ESC molecule (by forming the RAG–ESC complex). Notably, the RAG–ESC complex can also trigger DSBs at cryptic RSSs via a ‘*cut‐and‐run*’ reaction without the reintegration of the circular DNA molecule [11] (Fig. 1B). Gao *et al*. propose that this mechanism is responsible for the accumulation of additional mutations that fuel the early relapse in patients affected by a form of acute leukemia: B‐cell progenitor acute lymphocytic leukemia (BCP‐ALL). By analyzing whole genome sequencing data from BCP‐ALL patients, Gao *et al*. noticed a significant increase in structural variants at cRSS in patients who relapse compared with nonrelapsed patients. Crucially, some of these mutations affected genes known to be linked to leukemia relapse. Another important piece of evidence in support of their hypothesis is that the *RAG1* gene, which is normally silenced after V(D)J recombination is completed, remains detectable in BCP‐ALL patients, thus providing an opportunity for the ESCs to wreak havoc.

Distinct from ecDNAs, which confer a growth advantage and shape tumor progression mainly by mediating oncogene amplifications [12]. ESCs are thus proposed to contribute to tumor progression by promoting mutations of cancer‐driver genes and relapse‐associated genes primarily through ‘*cut‐and‐run*’ reactions. Since mutations are then inherited by all daughter cells, ESC maintenance might not be required over time, which explains the different magnitude between the number of ESCs (few copies in a subset of cells) and the number of ecDNAs (often hundreds of copies per cell in most cancer cells) detected in cancer patients.

These surprising results open a series of questions about the mechanisms and impact of this unique mutational process in cancers that future studies will have to answer. Are all ESCs equal, or are some of them better at inducing ‘*cut‐and‐run*’ mutations? Do all ESCs have the ability to replicate, or are some better than others? Do ESCs play analogous roles in other B‐cell neoplasms and in T‐cell tumors? The recent development of strategies to induce ecDNAs in a controlled manner has greatly helped the study of oncogene‐containing ecDNA contributions during tumorigenesis [13, 14]. Analogously, engineering the formation of ESCs in a controlled manner in primary and malignant B and T cells, alone or in combination with RAG1/2 expression manipulation, will be essential to establish a clear causal link between these small circular molecules and genome instability and tumor progression.

Gao *et al*.'s work is clinically relevant. ecDNA presence is a widespread phenomenon, being detected in approximately one‐quarter of cancers across different cancer types [12]. EcDNA status in tumors is associated with poor survival and increased tumor stage, thus suggesting a prominent role for ecDNAs in cancer progression and dissemination at distant sites. Gao *et al*. raise the possibility that ESCs can accumulate in additional subtypes of lymphocytic neoplasms, thus providing a novel prognostic tool to evaluate the risk of relapse in a broader range of cancer patients.

Oncogene‐containing extrachromosomal DNAs have emerged as a common class of cancer‐driver mutations; regardless of their origin and content, DNA circles provide cancer cells with a powerful substrate for adaptation and evolution. Unlike mutations on linear chromosomes, circular DNA elements are mobile, non‐Mendelian genetic elements, whose copy number can rapidly fluctuate in response to intrinsic and extrinsic selective pressures, thus allowing cancer cells to adapt to changing selective pressures coming from the tumor microenvironment or from therapeutic interventions. Whether extrachromosomal circles will ultimately prove to be equally prevalent drivers in B‐ and T‐cell malignancies remains uncertain, but it is now evident that these DNA elements warrant the full attention of the scientific community.

## Conflict of interest

The authors declare no conflict of interest.

## Author contributions

DP and AV conceived of and wrote this commentary.
